# Long-term integrated telerehabilitation of COPD Patients: a multicentre randomised controlled trial (iTrain)

**DOI:** 10.1186/s12890-016-0288-z

**Published:** 2016-08-22

**Authors:** Paolo Zanaboni, Birthe Dinesen, Audhild Hjalmarsen, Hanne Hoaas, Anne E. Holland, Cristino Carneiro Oliveira, Richard Wootton

**Affiliations:** 1 Norwegian Centre for E-health Research, University Hospital of North Norway, Tromsø, Norway; 2 Department of Health Science and Technology, Laboratory of Assistive Technologies - Telehealth & Telerehabilitation, SMI, Aalborg University, Aalborg, Denmark; 3 Heart and Lung Clinic, University Hospital of North Norway, Tromsø, Norway; 4 Department of Clinical Medicine, The Arctic University of Norway, Tromsø, Norway; 5 La Trobe University, Melbourne, Australia; 6 Alfred Health, Melbourne, Australia; 7 Institute for Breathing and Sleep, Melbourne, Australia

**Keywords:** COPD, Pulmonary rehabilitation, Telemedicine, Exercise, Home monitoring, Telerehabilitation

## Abstract

**Background:**

Pulmonary rehabilitation (PR) is an effective intervention for the management of people with chronic obstructive pulmonary disease (COPD). However, available resources are often limited, and many patients bear with poor availability of programmes. Sustaining PR benefits and regular exercise over the long term is difficult without any exercise maintenance strategy. In contrast to traditional centre-based PR programmes, telerehabilitation may promote more effective integration of exercise routines into daily life over the longer term and broaden its applicability and availability. A few studies showed promising results for telerehabilitation, but mostly with short-term interventions. The aim of this study is to compare long-term telerehabilitation with unsupervised exercise training at home and with standard care.

**Methods/Design:**

An international multicentre randomised controlled trial conducted across sites in three countries will recruit 120 patients with COPD. Participants will be randomly assigned to telerehabilitation, treadmill and control, and followed up for 2 years. The telerehabilitation intervention consists of individualised exercise training at home on a treadmill, telemonitoring by a physiotherapist via videoconferencing using a tablet computer, and self-management via a customised website. Patients in the treadmill arm are provided with a treadmill only to perform unsupervised exercise training at home. Patients in the control arm are offered standard care. The primary outcome is the combined number of hospitalisations and emergency department presentations. Secondary outcomes include changes in health status, quality of life, anxiety and depression, self-efficacy, subjective impression of change, physical performance, level of physical activity, and personal experiences in telerehabilitation.

**Discussion:**

This trial will provide evidence on whether long-term telerehabilitation represents a cost-effective strategy for the follow-up of patients with COPD. The delivery of telerehabilitation services will also broaden the availability of PR and maintenance strategies, especially to those living in remote areas and with no access to centre-based exercise programmes.

**Trial registration:**

ClinicalTrials.gov: NCT02258646.

**Electronic supplementary material:**

The online version of this article (doi:10.1186/s12890-016-0288-z) contains supplementary material, which is available to authorized users.

## Background

Chronic obstructive pulmonary disease (COPD) is characterised by a persistent airflow limitation that is usually progressive [1]. Patients experience frequent acute exacerbations, characterised by worsening of respiratory symptoms [2] which may lead, in the worst cases, to a hospital admission [3]. COPD poses a substantial burden on healthcare budgets. The largest part of the health service expenditure is for hospitalisations and emergency department (ED) presentations, which commonly occur in the latter stages of the disease [4]. Moreover, prior hospitalisations represent a risk factor for rehospitalisation in patients discharged after a severe exacerbation [5]. Hospital admissions for exacerbations do not only represent a burden for the healthcare system, but have also a negative impact on patients, who experience reduced physical activity, decreased exercise performance [6], and impaired quality of life, even in mild stages of the disease [7]. Dyspnoea, the most commonly reported symptom during acute events, is associated with anxiety and depression [8]. Importantly, reduced physical activity is the strongest predictor of mortality in patients with COPD [9].

Pulmonary rehabilitation (PR) is an evidence-based, multidisciplinary, and comprehensive intervention for the management of COPD [10–12]. The main goal is to improve the physical and psychological condition and to promote long-term adherence of health-enhancing behaviours [13]. Exercise training is the cornerstone of PR, aiming to improve physical capacity and ability to perform activities of daily living. Other components include patient assessment, behavioural change, education on self-management, and psychosocial support [13]. PR improves dyspnoea, physical performance, quality of life [10], and is effective in reducing use of healthcare resources [14]. However, the short-term benefits diminish over the succeeding 12 months without an effective maintenance strategy [15]. Sustaining long-term adherence to exercise training is difficult due to variation in day to-day condition, exacerbations, hospital admissions, and transportation problems [16]. Key factors which promote exercise maintenance include professional support, review of exercise intensity, goal setting, social support, positive personal attributes, and the availability of exercise programmes with regular supervision [16]. Only a few investigators have explored maintenance strategies to sustain the benefits of PR over the long term, with inconsistent results [17–20].

Telemedicine has the potential to improve access to PR and support long-term exercise maintenance strategies. Telerehabilitation is the use of information and communication technologies to provide rehabilitation services remotely to people in their homes or other environments [21]. In contrast to traditional centre-based programmes, undertaking PR within the home environment may promote more effective integration of exercise routines into daily life over the longer term [22]. Evidence of the use of telemedicine in PR is still limited [13]. A recent review showed that telemedicine was effective in increasing physical activity levels in patients with COPD [23]. A few studies, most of which uncontrolled, showed promising results for short-term telerehabilitation interventions in regards to feasibility, safety, exercise capacity, and health-related quality of life [24–29]. However, only one uncontrolled pilot study trialled a long-term telerehabilitation intervention for patients with COPD [30]. This study demonstrated positive outcomes in terms of exercise maintenance, physical performance, health status and quality of life [31]. Importantly, long-term adherence was supported by experienced health benefits, self-efficacy, emotional safety, and maintenance of motivation [32]. Larger controlled trials are needed to explore the long-term effects of telerehabilitation in COPD.

Developing new cost-effective ways to sustain regular exercise over the long term and broaden the applicability and availability of PR is an important goal in the COPD management. The aim of this study is to compare long-term telerehabilitation of COPD patients consisting of exercise training at home, telemonitoring, and self-management, with unsupervised exercise training at home and with standard care. We hypothesised that long-term telerehabilitation will reduce the number of hospital readmissions and improve patient’s level of physical activity, health status and quality of life. The results from this study will provide decision makers, as well as practitioners, evidence on whether telerehabilitation interventions might be added to the current offer of traditional PR programmes and maintenance strategies.

## Methods/Design

### Design

An international multicentre randomised controlled trial (RCT) conducted across sites in three countries (Norway, Australia, and Denmark), where 120 patients with COPD are randomly assigned to three arms (telerehabilitation, treadmill, control) in a 1:1:1 ratio and followed up for 2 years. The trial is restricted to patients who have volunteered and provided written informed consent in accordance with the Declaration of Helsinki. The trial received approval from the Regional Committee for Medical and Health Research Ethics in Norway (2014/676/REK nord), the Alfred Hospital Human Research Ethics Committee (289/14), and the North Denmark Region Committee on Health Research Ethics (N-20140038). The protocol of this RCT fulfils the Standard Protocol Items: Recommendations for Interventional Trials (SPIRIT) guidelines (Additional file 1).

### Eligibility criteria

To be eligible for enrolment, participants must have the following inclusion criteria: 1) a diagnosis of COPD, based on an FEV_1_/FVC ratio < 70 % [33], 2) moderate, severe or very severe airflow limitation, with forced expiratory volume in 1 s percentage (FEV_1_ % of predicted) < 80 %, 3) at least one COPD-related hospitalisation or COPD-related ED presentation in the 12 months prior to enrolment, 4) aged between 40 and 80 years, and 5) capable of providing signed written informed consent.

Participants are excluded if they have at least one of the following criteria: 1) attendance at a rehabilitation programme in the 6 months prior to enrolment; 2) participation in another clinical study that may have an impact on the primary outcome, 3) deemed by the healthcare team to be physically incapable of performing the study procedures, 4) presence of comorbidities which, in the opinion of the healthcare team, might prevent patients from safely undertaking an exercise programme at home (for example severe orthopaedic or neurological impairments, severe cognitive impairment), and 5) home environment not suitable for installation and use of rehabilitation and monitoring equipment. Potential participants are not excluded on the basis of their existing home Internet access, as this can be provided by the study.

### Randomisation

Randomisation is stratified by centre and disease severity (FEV_1_ < 50 % vs. FEV_1_ ≥ 50 %) to preserve homogeneity between arms with regard to severity of clinical status. Randomisation is web-based and performed via the WebCRF program developed by the Unit for Applied Clinical Research at the Norwegian University of Science and Technology in Trondheim. The tool uses a computerised block randomisation, and the size of the first, smallest and largest blocks is established based on the total number of patients expected to be included in the study. The randomisation sequence generated is concealed from the study team by the program. The same WebCRF program is used to fill out electronic case report forms (CRFs). This allows patient data from several centres to be entered into the same database.

### Interventions

#### Telerehabilitation arm

**Fig. 1:**
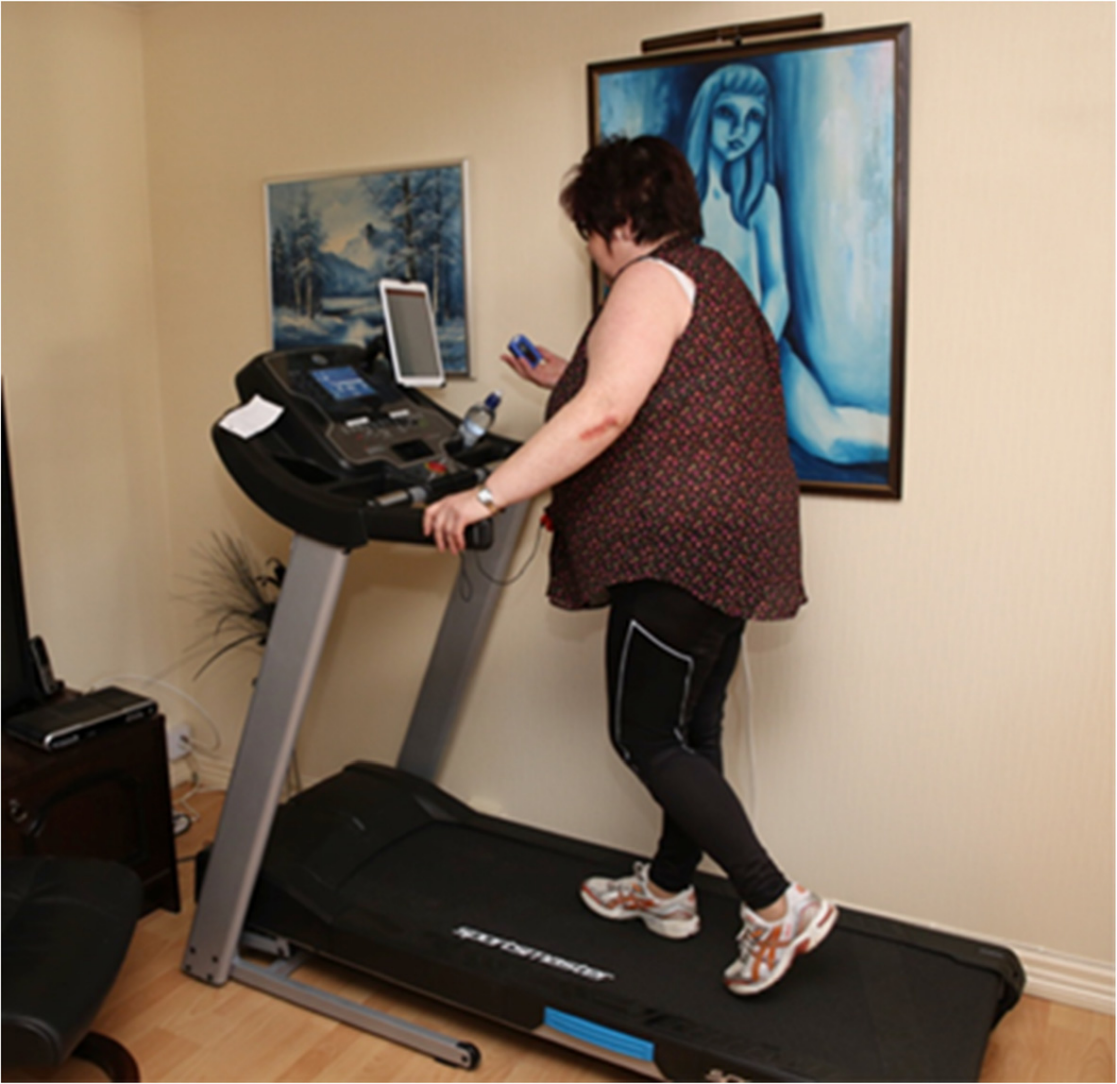
Telerehabilitation in a participant’s home

Patients in the telerehabilitation arm are offered an integrated intervention consisting of exercise training at home, telemonitoring, and self-management. The equipment includes: a) a treadmill (Sportsmaster T2 in Norway, Sportsmaster T3i in Denmark, Reebok ZRK1 in Australia), b) a pulse oximeter (Nonin 9570/9571), c) a tablet computer (Apple iPad Air), and d) a holder for the tablet computer (RAM) (Fig. 1). The feasibility of using such equipment was tested in a previous pilot study [30]. Videoconferencing is performed through Acano™ due to its ability to connect the participant’s tablet to videoconferencing protocol H.323 and Session Initiation Protocol (SIP) standards-based systems, desktop computers or mobile clients. Communication is performed with Advanced Encryption Standard (AES) encryption and features multiconferencing.

The exercise intervention consists of an individualised training programme of regular exercise of continuous or interval training on the treadmill and strength training exercises according to current guidelines [13]. The treadmill exercise programme lasts for at least 30 min. Depending on the patient’s condition, a programme of continuous training (moderate intensity - Borg scale [34] ratings up to 4) or interval training (high intensity - Borg scale ratings up to 6) is conducted. The frequency prescribed is 3–5 times/week for continuous training and 3 times/week for interval training. For interval training, up to 4 interval bouts of high intensity lasting from 1 to 4 min could be prescribed. Progression is made by increasing speed and incline first. Then the patient is encouraged to increase duration. Patients are permitted to take short rests if intolerable symptoms occur, but rest time does not count towards training duration. The exercise programme can be modified by the physiotherapist or the patients themselves according to their conditions. Strength training is prescribed for a frequency of 2–3 sessions per week. Each session includes at least two lower limb exercises (6–12 repetitions, 3 sets) and two upper limb exercises (6–12 repetitions, 1–3 sets). The patients can choose between sit-to-stand, squat, step-up, lunge, calf raise, biceps curl, shoulder press, wall push up, bench press, standing row, seated row, lateral pull down, triceps press. Bottles filled with sand/water or elastic bands/free weights can be used for upper limb exercises if already available in the patient’s home.

A customised website is used to access the individual training programme, fill in a daily diary and a training diary, review historical data, exchange electronic messages, schedule videoconferencing sessions, and assess individual goal settings and goal attainment. Patients are asked, every evening, to use their pulse oximeter at rest and to fill the daily electronic diary on the website including: a) oxygen saturation, b) heart rate, c) Breathlessness, Cough, and Sputum Scale (BCSS) [35], d) general wellbeing (qualitative self-score on a five-point Likert scale), and e) additional comments. During an exercise session on the treadmill, patients are required to self-monitor their oxygen saturation and heart rate. After each exercise session, patients are asked to fill in the electronic training diary on the website including: a) programme completion, b) Borg CR10 scale - Rating of Perceived Exertion for leg (highest value during exercise), c) Borg CR10 scale - Rating of Perceived Dyspnoea (highest value during exercise), d) oxygen saturation (lowest value during exercise), e) heart rate (highest value during exercise), and f) additional comments. The information sent through the electronic forms is monitored and interpreted weekly by a physiotherapist, who also checks for any out-of-range value. The physiotherapist can provide feedback to the patients via the website, or use this information in the upcoming videoconference. Patients are also informed on the presence of signs and symptoms to ensure that they do not exercise if not recommended.

Patients have scheduled videoconferencing sessions with the physiotherapist. During videoconferencing, patients are encouraged to set specific goals for their ongoing programme and their daily life activities. Self-management education and training are provided to promote adherence to health-enhancing behaviours. During disease exacerbations, strength training exercises might be encouraged until the patient gets well enough to exercise on the treadmill. If the patient reports changes on sputum aspect, guidance on airway clearance techniques is offered (e.g. active cycle of breathing). The physiotherapist can take electronic notes using the same website. The frequency of contacts consists of at least 1 individual videoconferencing session/week in the first 8 weeks after enrolment, and at least 1 individual videoconferencing session/month in the following period. Participants who experience a hospital admission during the study period are invited to continue their participation after discharge. In these cases, at least 1 individual videoconferencing session/week will be applied in the month after discharge as a reinforcement strategy. Additional peer-group exercise sessions supervised by the physiotherapist can be organised.

#### Treadmill arm

Patients in the treadmill arm are provided with a treadmill only to perform unsupervised exercise training at home. The exercise intervention consists of an individualised unsupervised training programme performed as prescribed to the participants in the telerehabilitation arm, without regular review or progression of the program. Participants are asked to record each training session on a paper-based diary. This intervention arm allows comparing the effects of providing training equipment only to those using telerehabilitation.

#### Control arm

Patients in the control arm are offered standard care. To ensure that no participants are denied access to the best health care practice, any participant in the trial can undertake a traditional PR programme at any time during the 2-year study period if it is considered clinically indicated by their usual treating team.

### Recruitment and study procedures

At enrolment, a clinical assessment is performed for all the patients by appropriately trained study personnel blinded to group allocation. Participants are asked to perform spirometry, the 6-min walking test [36] and complete the study questionnaires (Table 1). Participants are also provided brochures containing information about PR, physical activity and training, diet, self-management, motivation and lifestyle changes, smoking cessation and oxygen therapy.

**Table 1:** Data collected at baseline and follow-up visits

Data collected	Baseline	6-month	1-year	2-year
Clinical history and patient’s characteristics	X			
Pharmacological treatment	X			
Spirometry	X	X	X	X
6-min walking test	X	X	X	X
MMRC Dyspnoea Scale	X	X	X	X
COPD Assessment Test	X	X	X	X
EQ-5D Health Questionnaire	X	X	X	X
Generalised Self-Efficacy Scale	X	X	X	X
Hospital Anxiety and Depression Scale	X	X	X	X
Level of physical activity	X	X	X	X
Healthcare utilisation		X	X	X
Patient Global Impression of Change		X		

After enrolment, patients in the telerehabilitation and the treadmill arms undergo a supervised session on the treadmill with an experienced research physiotherapist to learn how to safely exercise at home, receive information on how to make progress in the programme and are given the opportunity to clarify any questions related to equipment management. Patients in the telerehabilitation arm also receive training on the use of the study website. A test videoconferencing session is also performed by the local research team to guarantee proper equipment functioning. Patients can always contact the research team in case of technical issues.

At 6-month, 1-year and 2-year, patients undergo a clinical reassessment and are asked to complete the study questionnaires (Table 1). During data collection patients are encouraged to continue participating in the study. Patients are discharged from the trial after 2 years, those in the telerehabilitation and the treadmill arms can keep the equipment at the end of the study.

### Outcome measures

The primary outcome is the combined number of hospitalisations and ED presentations. Differences in the rate of events between the study arms will be measured at all assessment time points (Table 2). Data on hospitalisations and ED presentations, together with outpatient visits, will be collected from hospitals records (in Australia), regional systems (in Denmark) and national registries (in Norway). Details will include institution, date of admission, date of discharge, diagnosis-related group (DRG), diagnoses, procedures and associated cost. In addition, use of hospital resources will be recorded during the follow-up visits. Hospitalisations and ED presentations will be also analysed separately as secondary outcomes. Mortality will be monitored along the study together with dropouts.

**Table 2:** Primary and secondary outcomes and related measures

Outcome	Measure
Primary outcome	
• Combined number of hospitalisations and ED presentations	• Incidence Density
Secondary outcomes	
• Hospitalisations • ED presentations • Mortality • Time free from first event • Health status • Quality of life • Anxiety and depression • Self-efficacy • Subjective impression of overall change • Physical performance • Level of physical activity	• Incidence Density • Incidence Density • Mortality rate • Days to first hospitalisation or ED presentation • COPD Assessment Test • EQ-5D • Hospital Anxiety and Depression Scale (HAD) • Generalised Self-Efficacy Scale (GSES) • Patient global impression of change scale (PGIC) • 6-min walking distance (6MWD) • Daily number of steps, minutes of moderate to vigorous physical activity and sedentary time during 1-week
• Cost-effectiveness • Experiences in telerehabilitation	• Cost-utility analysis (cost-per-QALY) • Qualitative interviews with semi-structured questions

Health status will be measured with the COPD Assessment Test (CAT) [37]. Health-related quality of life will be measured with the EQ-5D questionnaire [38]. Levels of anxiety and depression will be measured with the Hospital Anxiety and Depression Scale (HAD) [39]. Self-efficacy will be measured with the Generalised Self-Efficacy Scale (GSES) [40]. Subjective impression of overall change will be measured with the Patient global impression of change scale (PGIC) [41]. The study questionnaires, available in English, Norwegian and Danish with validated versions, will be collected along the study at different time points (Table 1).

Functional exercise capacity will be measured with the 6-min walking distance (6MWD). The 6-min walking test will be performed twice according to guidelines, and the furthest distance recorded [36].

Objective physical activity assessment will be undertaken using the SenseWear Armband (SWA; BodyMedia, Pittsburgh, USA; professional software version 7.0). The SWA will be positioned on the participant’s left upper arm according to manufacturer instructions. Participants will be instructed to wear the SWA for 1 week, only removing it for bathing or water-based activities. The first and last days of data will be excluded from analysis upon data retrieval. A day of data (midnight to 23:59) will be included for analysis if there is at least 10 h of data within the 24-h period. A minimum of four valid days of data will be required per participant at each assessment time point [42], inclusive of at least 1 weekend day. The proprietary algorithm provides a range of variables for each minute of wear time, including energy expenditure and number of steps. The intensity of physical activity is described according to metabolic equivalents (1 MET = 1 kcal/kg/h). Each minute of wear time will be allocated to a category of physical activity on the basis of MET classification (sedentary ≤ 1.5 METs, moderate and vigorous ≥ 3 METs). The amount of time spent in moderate to vigorous physical activity, and time spent sedentary, will be calculated.

A cost-utility analysis will be performed to verify whether the telerehabilitation and the treadmill interventions are cost-effective. The perspective of the healthcare authority will be adopted with respect to costs. The following cost components will be included in the analysis: a) hospital resources, b) delivery of the telerehabilitation intervention, and c) equipment. Hospital resources will include hospitalisations, ED presentations, outpatient visits, and rehabilitation. Unit cost for each resource will be based on the specific public tariffs from the national DRG system. Delivery of the telerehabilitation intervention will include the time used by the project team to install the equipment in the patient’s home and provide training, and the time used by the physiotherapist to supervise the patients. Equipment costs for the telerehabilitation arm and the intervention arm will be calculated based on a 5-year amortisation period. All costs will be expressed in Euros (€). Utility will be measured in terms of quality adjusted life years (QALYs), based on the answers of the EQ-5D questionnaires at baseline, 6 month, 1–2 years. Utility values will be calculated using the European EQ-net VAS set, and only if all the five dimensions are answered. The incremental cost-effectiveness ratio will be computed as differential costs and differential QALYs.

Patients’ perspectives in participating in long-term telerehabilitation will be explored through qualitative interviews with semi-structured questions. Interviews will be conducted before telerehabilitation, after 1 year and after 2 years, with 5–8 patients in the telerehabilitation arm at each site. Interviews will be recorded on audio digital file, transcribed verbatim and analysed via Nvivo 10.0 upon the theoretical frame of the learning theory [43].

### Adverse events and dropouts

Adverse events, including deaths, treadmill injuries and other unspecified reasons, will be recorded in the WebCRF program. Technical problems will be recorded by each participating centre into a separate database. Dropouts will occur if patients notify the research team that they do not want to participate any longer to the study and therefore withdraw their consent. In this case the project team might collect the equipment upon request. Patients who do not participate actively in the intervention will still be included in the study, and analysed according to the intention-to-treat approach. They will keep the project equipment as long as they want.

### Statistical analysis

Descriptive statistics at baseline will be reported as means ± SD for normally distributed continuous variables, or medians with 25–75th percentiles in the case of skewed distribution. Normality of distribution will be tested by means of the nonparametric Kolmogorov-Smirnov test. An intention-to-treat analysis will be performed on all randomised subjects to provide unbiased comparisons among groups and avoid the effects of dropout. The primary outcome and related secondary outcomes will be measured through the Incidence Density, defined as the number of events in a group divided by the total person-time accumulated during the study in that group [44]. Differences between study arms will be tested by the Comparison of Incidence Rates. A two-sided test and a significance level of *α* = 0.05 will be used. All events from the day after randomisation to patient exit/death will be included. Other secondary outcomes will be measured as changes from baseline to all assessment time points. Changes of the secondary outcomes will be tested by use of linear mixed models, which allow accounting for repeated measures collected in a longitudinal design. Moreover, linear mixed models deal better with dropouts that other methods used for repeated measures, and use of imputation techniques for missing data is not necessary. A *p*-value <0.05 will be considered significant for all tests. All statistical analyses will be performed by using IBM SPSS Statistics.

### Sample size

The sample size requirements for this study were intended to provide adequate power for the analysis of the primary outcome. From studies with patients with similar characteristics [45–48], we estimated an incidence density used as a null hypothesis of 2 events per person-year, and a 40 % relative reduction in the primary outcome. In a major study of a management programme including home exercises for COPD patients after acute exacerbations [46] the mean number of hospital admissions per patient was reduced from 1.6 to 0.9 in the year following a hospital admission. We calculated that a sample size of 65 person-years per group would allow a power of 95 % to detect an incidence rate ratio of 0.60, with a type-I error (α) of 0.05. Assuming that up to 20 % of patients may drop out uniformly over the intervention period, 40 patients (corresponding to 80 person-years) will be enrolled per each of the three arms for the 2-year study duration. In total, 120 patients (corresponding to 240 person-years) will therefore be enrolled in the study. Recruitment is expected to be concluded by the end of 2016.

## Discussion

This study protocol described the methods used in this first RCT investigating the effects of long-term telerehabilitation in COPD. Patients with COPD often experience repeated exacerbations which lead to a worsening of their health condition and to hospital admissions [3]. Moreover, hospital readmissions are more likely to occur in patients with prior history of hospitalisations [5]. There is an increasing need for cost-effective treatment strategies for patients with COPD [22]. PR is a low-cost, integral component of COPD management [12]. PR is traditionally centre-based and offered either as a 6–12-week outpatient programme or as a 4-week inpatient programme. Available resources for PR are often limited, and patients living in rural areas especially suffer from poor availability of PR programmes [49]. Maintenance strategies are needed to sustain the benefits of PR over the long term [15]. However, these are scarcely documented, and the optimal combination of maintenance interventions after completion of a PR programme remains unknown [12]. Long-term telerehabilitation is an innovative intervention which might reduce hospital readmissions in COPD and thus limit healthcare utilisation. The results of this study will provide evidence on whether long-term telerehabilitation represents a cost-effective strategy for the follow-up of patients with COPD. The delivery of telerehabilitation services will also broaden the availability of PR and maintenance strategies, especially to those living in remote areas and with no access to centre-based exercise programmes.

Significant improvements in outcomes can be obtained with both supervised and unsupervised home exercise [50]. However, sustaining long-term exercise is generally difficult [17] for many reasons, including professional support, regular supervision [16], emotional safety, and maintenance of motivation [32]. We expect that standard care and unsupervised home exercise will be less beneficial than a telerehabilitation intervention where patients are supervised regularly by a physiotherapist via videoconferencing. The study was designed as a three-armed RCT to isolate the effects of placing training equipment in the patient’s home from those of telemonitoring.

### Trial status

Patient recruitment commenced in October 2014 and is continuing.

## Additional file

Additional file 1: SPIRIT checklist. (DOC 121 kb)

## Abbreviations

6MWD: 6-minute walking distance
AES: Advanced encryption standard
BCSS: Breathlessness, cough, and sputum scale
CAT: COPD Assessment test
COPD: Chronic obstructive pulmonary disease
ED: Emergency department
GSES: Generalised self-efficacy scale
HAD: Hospital anxiety and depression scale
PGIC: Patient global impression of change scale
PR: Pulmonary rehabilitation
RCT: Randomised controlled trial
SIP: Session initiation protocol
